# Editorial: Critical complications in pediatric oncology and hematopoietic cell transplant, volume III

**DOI:** 10.3389/fonc.2026.1991554

**Published:** 2026-09-23

**Authors:** Dristhi Ragoonanan, Jennifer Ann McArthur, Asya Agulnik, Marie E. Steiner, Kris M. Mahadeo

**Affiliations:** 1 Division of Pediatric Hematology Oncology, Cure 4 the Kids Foundation, Las Vegas, NV, United States; 2 Division of Critical Care Medicine, Department of Pediatrics, St Jude Children’s Research Hospital, Memphis, TN, United States; 3 Department of Pediatric Global Medicine, St Jude Children’s Research Hospital, Memphis, TN, United States; 4 Division of Pediatric Hematology Oncology and Division of Pediatric Critical Care, Department of Pediatrics, University of Minnesota, Minneapolis, MN, United States; 5 Division of Pediatric Transplantation and Cellular Therapy, Duke University School of Medicine, Durham, NC, United States

**Keywords:** extracoporeal life support, multidisciplinary approach, pediatric critical care, pediatric hematopoietic cell transplantation (HCT), pediatric oncology

## Summary of volume I and II

The role of critical care (CC) in pediatric oncology and hematopoietic cell transplant (HCT) patients continues to evolve and expand. In its infancy, outcomes for these patients requiring intensive care was dismal, with mortality rates exceeding 80% (1, 2). Additionally critical care interventions, including renal replacement therapy and mechanical ventilation, were associated with a higher risk of death and carried a near total fatality rate (1, 3). As a result, CC and intensive interventions were often withheld due to perceived futility. However, over the past two decades, there has been a steady decline in HCT patient mortality among those requiring critical care. In a large series combing data from the Center for International Blood and Marrow Transplant Research(CIBMTR) and the Virtual Pediatric Systems (VPS), Zinter et al. reported an initial intensive care unit (ICU) survival rate of 85.7% in 6995 patients undergoing their first allogeneic HCT (4). Despite the initial high ICU discharge rate, however, more than half of these patients required ICU readmission resulting in a 1 year overall survival (OS) of 52.5%. These findings highlight that although ICU discharge rates have significantly improved, substantial challenges remain in optimizing long-term care and reducing the risk of readmission.

Given the room for continued improvement in patient outcomes for this vulnerable population, this current Research Topic, *Critical Complications in Pediatric Oncology and Hematopoietic Cell Transplant Volume III* builds upon the prior themes of Volumes I and II. Combined, these three volumes of this research topic contain a total of 72 publications representing 33 countries from around the world, reflecting a broad international and multidisciplinary perspective on the care of critically ill pediatric oncology and HCT patients (**Figure 1**).

**Figure 1:**
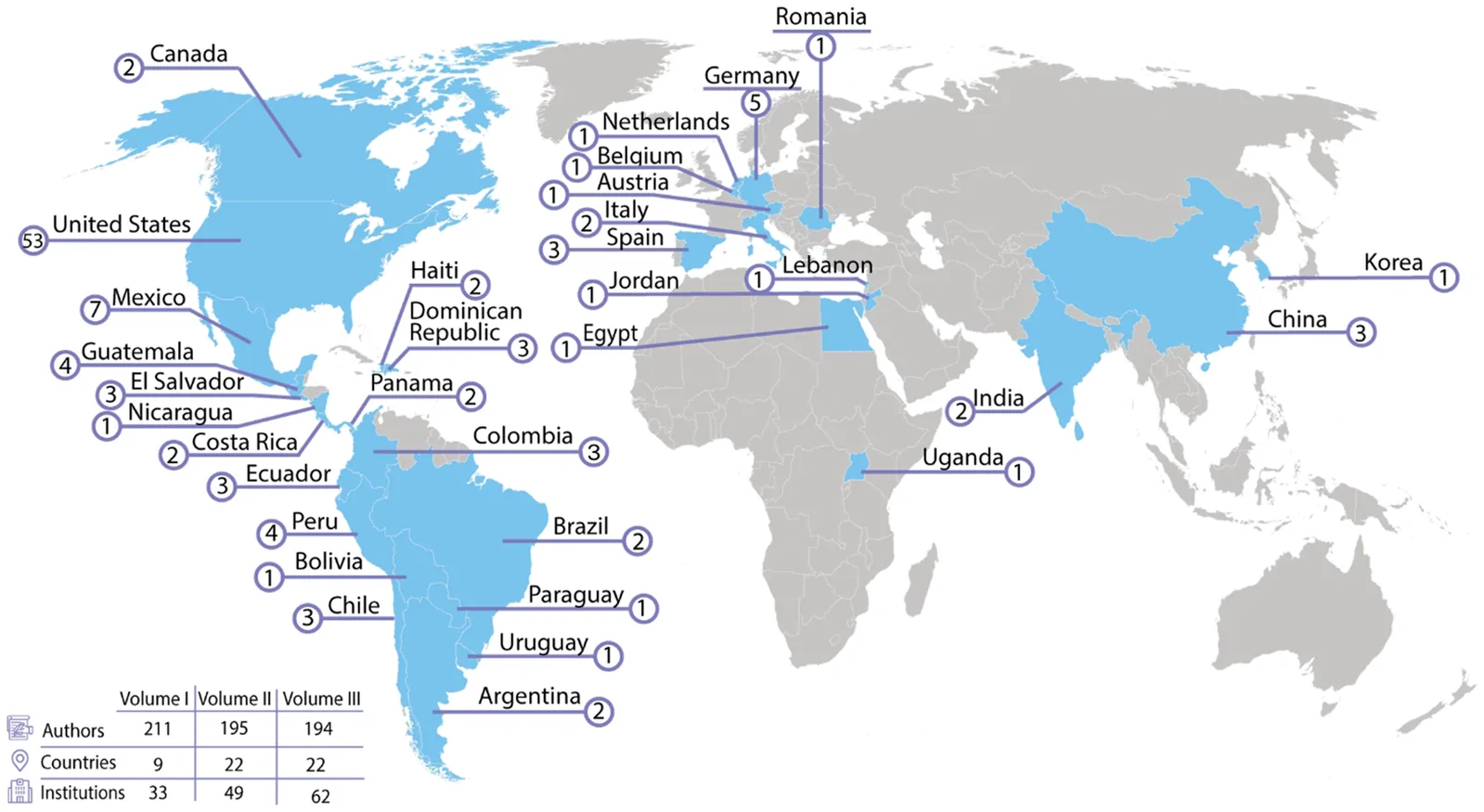
Evidence for growing international interest in the field of pediatric onco-critical care.

In the first two volumes, publications addressed 1) predictive models to better identify patients at risk for requiring critical care support 2) strategies to improve the early recognition of clinical deterioration and timely escalation of care 3) outcomes among patients requiring pediatric ICU (PICU) support, proving that intensive care interventions are not futile and can provide meaningful benefit 4) strategies to better recognize, reduce and manage complications post HCT 5) the evolving use of critical care interventions including mechanical ventilation, renal replacement therapy, and extracorporeal membrane oxygenation (ECMO), many of which were historically withheld for patients post HCT 6) the importance of multidisciplinary collaboration and communication and 7) global applicability of advances in critical care and the need for adaptations for different healthcare resource settings.

Volume III of the *Research Topic, Critical Complications in Pediatric Oncology and Hematopoietic Cell Transplant*, further emphasizes strategies to improve outcomes through earlier recognition of clinical deterioration and complications, timely intervention and advancing the boundaries on utilization of advanced life sustaining therapies. Importantly, Volume III also expands the focus beyond acute ICU survival to longer term outcomes, an area in which significant knowledge gaps remain.

## Rethinking futility: improving outcomes through critical care

Despite many advances, survival of pediatric patients with cancer and/or those undergoing HCT who require CC remains lower as compared to other pediatric patients requiring CC (5). Yet, outcomes may be nuanced among specific sub-populations of pediatric oncology/HCT patients. While the need for CC intervention for pediatric patients undergoing HCT is high, it is not in vain. Infants particularly are at disproportionately higher risk for transplant-related morbidity and mortality due to immature and developing organ systems. Alqathani et al. reported a high rate of CC resource utilization among 107 infants undergoing HCT; 41% required CC support and 56.8% of which required invasive mechanical ventilation. While infants undergoing HCT have a disproportionately higher risk of requiring CC compared to older pediatric patients, OS was high. Importantly, those who underwent HCT after 2013, had a significantly (59%) lower risk of mortality compared to those transplanted before 2013^4,6^. While ICU admission rate and associated mortality were higher than patients not admitted to the ICU (HR = 3.59), and despite the high rate of ICU admission, for those who survived “there were no clear differences in one-year post-HCT complication rates between infants who required CC support and those that did not.” (6) These findings highlight the impact of age and age-specific HCT primary disease indications on outcomes as well as the importance of considering advancements in HCT techniques, CC, and supportive care during prognostication. The findings also suggest that infants should be supported as aggressively as older pediatric HCT patients despite higher rates of CC resource utilization.

The complexity of critically ill pediatric oncology and HCT patients continue to increase, with outcomes increasingly influenced by comorbidities and organ dysfunction beyond their primary diagnosis. Lenz et al. described this evolving complexity, reporting that between 2001-2019, the prevalence of chronic non-oncologic complications among ICU patients rose from 44.3% to 69.1% for oncology patients and from 41.4% to 81.5% for HCT patients. This increasing comorbidity burden was accompanied by a marked rise in multiple organ dysfunction syndrome (MODS), which more than tripled for both oncology (9.5% to 33.3%; RR 3.52) and HCT (12.4% to 39.7%; RR 3.20) patients during this period.

The rising prevalence of multiorgan dysfunction has been accompanied by an evolution in the candidacy for advanced organ support modalities such as mechanical ventilation, continuous renal replacement therapy (CRRT) and ECMO for this patient population. Historically the use of extracorporeal life support (ECLS), particularly ECMO in HCT patients, was often considered futile due to poor outcomes. However, an analysis of the Extracorporeal Life Support Organization’s (ELSO) registry from 1990–2020 demonstrated not only an increasing utilization of ECLS among pediatric patients, but also meaningful improvements to hospital discharge over the past decade, rising from 5-10% to 26% (6).

Despite these advancements, assessing candidacy for ECMO remains challenging. In a retrospective study of 19 pediatric patients, Belevskaia et al. reported a 100% mortality of the 4 HCT patients receiving ECMO, while more promisingly, three of the five oncology patients survived. Other more established components of critical care such as mechanical ventilation, while more widely used is also associated with an increased risk of mortality as reported in this volume by authors Alqathani et al., Kim et al., Lenz et al. and Lasso et al.

These findings highlight knowledge gaps in our current understanding of critical care interventions for this vulnerable population and the need for dedicated multidisciplinary teams to help guide decision making. This concept is nicely outlined by Hurley at al. in the article “New perspectives on extracorporeal life support: expert teams and precise selection of candidates are transforming pediatric cancer and hematopoietic cell transplantation care.

## Bridging borders: advancing critical care across resource-variable settings

Despite observed improved outcomes for critically ill pediatric oncology and HCT patients in high-resource settings, it remains challenging to translate these successes to lower- resource hospitals where differences in healthcare infrastructure and unique socioeconomic barriers exist.

Lasso et al. reported in their 9-year study of pediatric HCT patients (<18 years) in Colombia, that the primary cause of pediatric ICU admission was infection in 68.1% of cases. Nearly half of these patients (49.3%) had confirmed bacteremia, a substantial proportion of which was due to multidrug resistant organisms. Additionally, sepsis accounted for 66.6% of deaths and 100-day post HCT survival rate was 51.6% (11). These findings contrast with those reported in higher resources settings. Zinter et al. analyzed data from the VPS and CIBMTR databases between 2009 and 2014 in the United States and reported a far lower PICU mortality rate of 17.5% in pediatric patients after allogeneic HCT (7). In another study included in Volume III, Goel et al. conversely reported respiratory decline (73%) not infection as the highest indication for admission and a 100-day pediatric ICU mortality rate of 25%. Together these studies highlight important global differences and the need to adapt advances in critical care to best suit each patient population and healthcare setting.

One potential method of bridging healthcare disparities is understanding how proven evidence-based interventions can be effectively adapted and implemented. Quijano-Lievano et al. evaluated the implementation of Escala de Valoración de Alerta Temprana (EVAT), a Spanish-language Pediatric Early Warning System (PEWS), in six variable resource hospitals in South America and Europe caring for pediatric patients undergoing HCT. Although the reduction in clinical deterioration event (CDE) mortality post PEWS implementation did not reach statistical significance (24% vs 15%), earlier recognition of clinical deterioration was observed. CDE post-PEWS were less likely due to respiratory distress (38% *vs* 23% *p=*0.0348), had fewer organ systems with dysfunction at ICU transfer (median 2 *vs*. 0, *p<0.*001), and had lower severity of illness at ICU transfer. Complementing these findings, Sijecic et al. also reported on the provider perspective on implementation of PEWS in 58 Latin American hospitals and reported perceived multilevel benefits for pediatric oncology patients. Together, these studies highlight the unique challenges faced by different health care settings. They demonstrate that evidence proven interventions such as the use of the established PEWS and other evidence-based interventions are both feasible and beneficial, and may help to narrow global disparities and potentially improve outcomes.

## Recognition to prediction

Pediatric oncology patients and those undergoing HCT represent a unique, medically complex population vulnerable to a multitude of complications secondary to both their underlying disease and ongoing treatment. The ability to predict which patients are at highest risk for life threatening complications has the potential to rapidly identify vulnerable patients, guide treatment decisions and potentially provide novel treatment targets. Endotheliopathies (injury and dysfunction of the endothelium) in particular, can result not only in life-threatening complications post HCT, such as sinusoidal obstruction syndrome/veno-occlusive disease, graft vs host disease, transplant associated thrombotic microangiopathy, idiopathic pneumonia syndrome but also impaired graft function and delayed hematopoietic recovery. Uchida et al. report potential biomarkers of glycocalyx damage and dysregulation studied at both St Jude Children’s Research Hospital and MD Anderson Cancer Center. This noninvasive blood test reported Syndecan-1 and Angiopoietin-2 as promising potential biomarkers with consistent strong predictive power to detect endotheoliopathy (17).

Ghafoor et al. also sought to assess the potential utility of Endothelial Activation and Stress Index (EASIX) and its modified version (m-EASIX) as predictive markers of endothelial complications in pediatrics. These models use composite scores derived from routine lab parameters and have been studied primarily in adult populations. Although the study included only 31 patients, EASIX and m-EASIX scores were significantly higher in children with endothelial complications than in controls. (p = 0.01) at Day 21 Importantly, detection of endotheliopathies at Day+ 21 may represent a relatively late indicator of endothelial injury underscoring the need to identify earlier and more reliable predictors.

## Advancing the next frontier

The incidence of childhood cancer as well as the number of HCT’s performed in the US have increased over the past decades with encouraging improvement in both short- and long-term survival (8, 9). Pediatric oncology and HCT patients however requiring CC continue to experience disproportionately higher morbidity and mortality than the general pediatric patients. Alcamo et al. reported pediatric oncology patients treated for sepsis had an increased mortality or new morbidity than non-oncology patients (22.7% vs 12.1%). As survival from childhood cancer and HCT continue to improve, attention must also extend beyond acute survival to include the long-lasting physical and mental effects of critical illness. Kroupina et al. highlight the mental health care needs of these patients and propose early identification of stressors and early implementation of targeted supportive interventions.

Further work is also needed to better define and characterize organ dysfunction in pediatric oncology and HCT patients as their underlying disease and unique pathophysiology may limit the applicability of criteria designed for the broader pediatric population and adults. Addressing this gap, Bashir et al. describe a proposed modification of The Pediatric Organ Dysfunction Information Update Mandate (PODIUM) for pediatric oncology and post-HCT patients (PODIUM-Onc). This international, multi-disciplinary effort seeks to establish consensus criteria through comprehensive evaluation of published global literature, multidisciplinary discussion among various specialties and the unique input of pediatric cancer care survivors.

In summary, Volume III of *Critical complications in pediatric oncology and hematopoietic cell transplant* continues to shape the future of pediatric critical care for oncology and HCT patients. From establishing population specific scoring systems to potentially earlier identification of critical complications to challenging historical boundaries for extracorporeal life support to reducing long term morbidity, this volume adds to the growing body of literature that critical care in in pediatric oncology and HCT population is far from futile and that patients can not only survive with CC but thrive.
